# Copper-coated hospital surfaces: reduction of total bacterial loads and resistant *Acinetobacter* spp.

**DOI:** 10.1186/s13568-022-01491-x

**Published:** 2022-11-23

**Authors:** Cláudia Justin Blehm, Marina Silveira Gregis Monteiro, Marjo Cadó Bessa, Mariana Leyser, Amanda Simão Dias, Juliana Sumienski, Stephanie Wagner Gallo, Anelise Baptista da Silva, Andressa Barros, Roberta Marco, Camila Piuco Preve, Carlos Alexandre Sanchez Ferreira, Fabiano Ramos, Sílvia Dias de Oliveira

**Affiliations:** 1 Laboratório de Imunologia e Microbiologia, Escola de Ciências da Saúde e da Vida, Pontifícia Universidade Católica do Rio Grande do Sul, PUCRS, Porto Alegre, RS Brazil; 2 Serviço de Controle de Infecção e Infectologia, Hospital São Lucas, Pontifícia Universidade Católica do Rio Grande do Sul, PUCRS, Porto Alegre, RS Brazil; 3 Programa de Pós-Graduação em Biologia Celular e Molecular, Escola de Ciências da Saúde e da Vida, Pontifícia Universidade Católica do Rio Grande do Sul, PUCRS, Porto Alegre, RS Brazil

**Keywords:** Healthcare-associated infection, *Acinetobacter* spp., Antimicrobial copper, Hospital environment, Copper coating

## Abstract

Healthcare-associated infections (HAIs) represent a global challenge and an even more staggering concern when related to microorganisms capable of resisting and surviving for long periods in the environment, such as *Acinetobacter* spp. Strategies that allow a reduction of pathogens from hospital environments represent an additional barrier in infection control protocols, minimizing transmission to hospitalized patients. Considering the antimicrobial properties of copper, here, the bacterial load and the presence of *Acinetobacter* spp. were monitored on high handling surfaces covered by 99.9% copper films on intensive and non-intensive care unit bedrooms in a tertiary care hospital. Firstly, copper-coated films were able to inhibit the adhesion and biofilm formation of *A. baumannii* strains in in vitro assays. On the other hand, *Acinetobacter* spp. were isolated from both copper-coated and uncoated surfaces in the hospital, although the majority was detected on surfaces without copper. All carbapenem-resistant *A. baumannii* isolates identified harbored the *bla*_oxa-23_ gene, while the *A. nosocomialis* isolates were susceptible to most antimicrobials tested. All isolates were susceptible to polymyxin B. Regarding the total aerobic bacteria, surfaces with copper-coated films presented lower total loads than those detected for controls. Copper coating films may be a workable strategy to mitigate HAIs, given their potential in reducing bacterial loads in nosocomial environments, including threatening pathogens like *A. baumannii*.

## Introduction

Healthcare-associated infections (HAIs) represent a global challenge and contribute to increasing hospitalization length stay, costs, and morbidity and mortality rates (Stone 2009; Allegranzi et al. 2011). HAIs are an even utmost concern when caused by agents harboring antimicrobial resistance determinants and capable of surviving for long periods of time in the environment (Kramer and Assadian 2014; Friedrich 2019). In this context, *Acinetobacter* spp., especially *A*. *baumannii*, stand out due to their ability to interact with abiotic surfaces and promote biofilm formation, hindering, therefore, the action of antimicrobial agents (Tomaras et al. 2003; Bardbari et al. 2017; Eze et al. 2018). Moreover, *Acinetobacter* spp. present an extensive genetic plasticity, which contribute to their success in infection, as well as in the ability to survive the highly selective pressure exerted by antimicrobials in nosocomial environments (Watkins and Bonomo 2016; Traglia et al. 2018). The ultimate consequence of this scenario is that *A. baumannii* corresponds to one of the most prevalent HAIs-related agents (CDC 2019; ECDC 2019, 2020; ANVISA 2020).

Persistence of multidrug-resistant *Acinetobacter* spp. on hospital surfaces and their resistance to cleaning protocols that utilize conventional sanitizers have been associated with outbreaks that are difficult to overcome (Strassle et al. 2012; Teare et al. 2019). In intensive care units (ICUs), persistence of these pathogens increases the risk of infection, due to the severity of clinical conditions that affect hospitalized patients usually submitted to highly invasive procedures (Borges Duarte et al. 2022). Several surfaces surrounding colonized and infected patients have been found contaminated with *Acinetobacter* spp., such as supply carts, floors, bed rails, bedside tables, infusion pumps, medical devices, and sinks (Wang et al. 2003; Thom et al. 2011; Raro et al. 2017). Measures of infection prevention and control (IPC), including active surveillance, contact precautions, staff training on cleaning and hand washing procedures, and antimicrobial stewardship, are needed to prevent outbreaks (Tacconelli et al. 2014; Karampatakis et al. 2019). Despite the efforts, however, the effectiveness in controlling carbapenem-resistant *A*. *baumannii* infections is often insufficient (Tacconelli et al. 2014).

A strategy to improve IPC measures and control the spread of hospital pathogens includes the use of metallic nanoparticles, especially copper and silver, covering surfaces and medical devices (Ruparelia et al. 2008; Palza 2015; Montero et al. 2019). Existing both in metallic and ionic forms, copper often alternate between cuprous and cupric oxidation states, which are usually the most harmful states to bacteria (Popov et al. 2020). Although copper is required in trace amounts for bacterial growth, enabling interaction with molecular oxygen, it is also toxic when present in excess, due to the production of reactive oxygen species through Fenton-like reactions (Mikolay et al. 2010; Popov et al. 2020). The mechanism of action of ionic copper includes rupture of bacterial cell membrane, osmotic imbalance, oxidative damage, and DNA deterioration (Borkow and Gabbay 2005; Grass et al. 2011).

Surface coatings with antibacterial materials, such as copper, have been investigated, demonstrating reduction of bacterial counts compared to untreated control surfaces (Faúndez et al. 2004; Casey et al. 2010; Inkinen et al. 2017), and indicating, therefore, the potential of copper in preventing bacterial transmission from contaminated surfaces to patients. In a clinical trial, incidence rates of HAIs were reduced by 58% in patients hospitalized in bedrooms whose surfaces were coated with copper when compared to patients in bedrooms without copper (Michels et al. 2015). Therefore, we evaluated the in situ contribution of 99.9% copper-coated films adhered to different surfaces of ICU and non-ICU bedrooms aiming to decrease the total aerobic bacteria loads, but with special concern for *Acinetobacter* spp. Additionally, the antimicrobial susceptibility of *A*. *baumannii* and *A*. *nosocomialis* isolates recovered from surfaces coated or uncoated with copper was characterized.

## Material and methods

### In vitro evaluation of copper film against *A. baumannii*

The in vitro antimicrobial efficacy of adhesive copper-coated films was evaluated using the reference strain *A. baumannii* ATCC 17978 and the clinical isolate *A*. *baumannii* Acb-220, previously classified as extreme drug-resistant (XDR), harboring *bla*_OXA-23_ gene and able to form biofilm in polystyrene surface (unpublished observations). Electrolytic Tough Pitch 99.9% copper-coated adhesive films with 1 cm^2^ and thickness of 50 µm were provided by CUNOV^®^ (Chile), and sterilized by ethylene oxide. The films were supplied as adhesives with 9.5 N/cm adherence to steel, tensile strength of 50 N/cm and high temperature resistance (ranging from − 10 °C to + 120 °C).

**Fig. 1:**
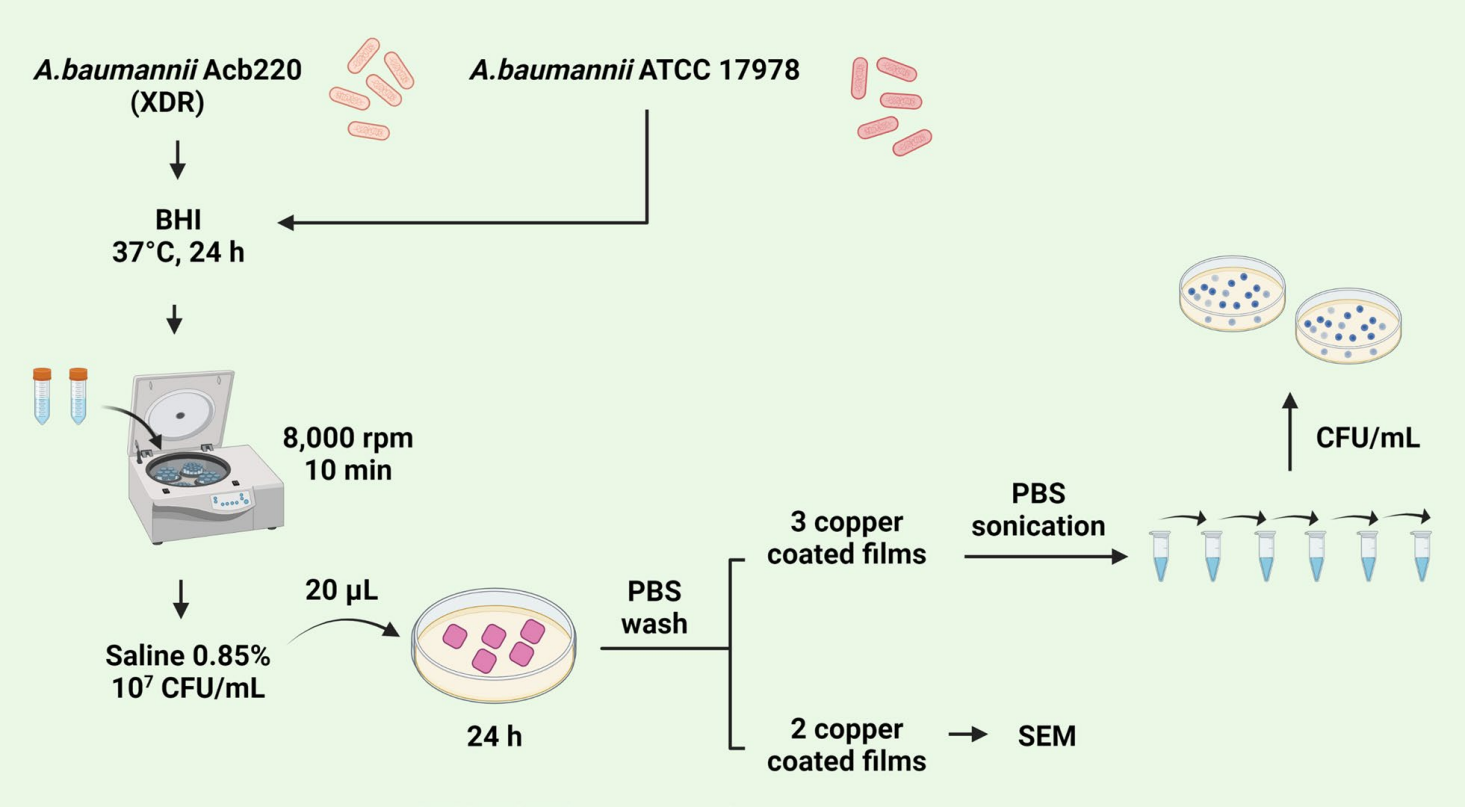
Flowchart of in vitro evaluation of copper film against *A. baumannii* ATCC 17978 and XDR *A. baumannii* Acb-220

Bacterial cultures were grown overnight at 37 °C in Brain Heart Infusion (BHI) broth (Oxoid, England), centrifuged at 8,000 rpm for 10 min, resuspended in 0.85% saline solution (Labsynth, Brazil) and adjusted to a cell density of 10^7^ colony-forming units (CFU)/mL. A 20 µL aliquot of adjusted inoculum was placed in Petri dishes, covered with five samples of 1 cm^2^ sterile copper-coated films and incubated at room temperature for 1 h to evaluate bacterial adhesion. Afterwards, the films were gently washed three times with sterile phosphate-buffered saline (PBS) to remove weakly adhered bacteria. Out of the five copper-coated films, three were sonicated with 10 mL of PBS and the detached cells were serially diluted until 10^–5^, being 100-µL aliquots spread on nutrient agar (Oxoid, England) and incubated for 24 h at 37 °C. The other two copper-coated films were analyzed by scanning electron microscopy (SEM) (Fig. 1). All assays were performed on triplicate. The same procedure was conducted with the clinical isolate Acb-220 to evaluate biofilm formation ability. After 1 h and 24 h incubation, copper-coated films were carefully removed from the culture medium, washed with PBS and fixed with 2.5% glutaraldehyde. Films were then dehydrated with increasing concentrations of acetone (30–100%), desiccated and metallized with gold. Samples were analyzed by SEM using a secondary electron detector at 15.0 kV (INSPECT-F50, FEI) at the Central Laboratory of Microscopy and Microanalysis (LabCeMM), PUCRS.

### Uncoated and copper-coated surface sampling on hospital bedrooms

Antimicrobial activity of copper-coated films was evaluated in six bedrooms of a 603-bed tertiary care hospital in Porto Alegre, Brazil, from December 2018 to May 2019. Two bedrooms of ICU and four of non-ICU were monitored. Three bedrooms received copper-coated films (CUNOV^®^, Chile) and three bedrooms (all of non-ICU) were used as control without surface coverage. Since not all sites were present in every ICU and non-ICU rooms, different surfaces were sampled (Table 1).

**Table 1:** Bedroom surfaces from Intensive Care Units (ICU) and non-ICU inpatient units sampled in this study

Two ICU bedrooms	Four non-ICU bedrooms
Shower troley	Light switches
Medication prep table	Door handles
Light switches	Sink faucet handles
	Bed rails
	Bed crank
	Over-bed tray table
	Toilet support rails
	Toilet flush buttons
	Toilet seats

### Total aerobic bacterial loads

**Fig. 2:**
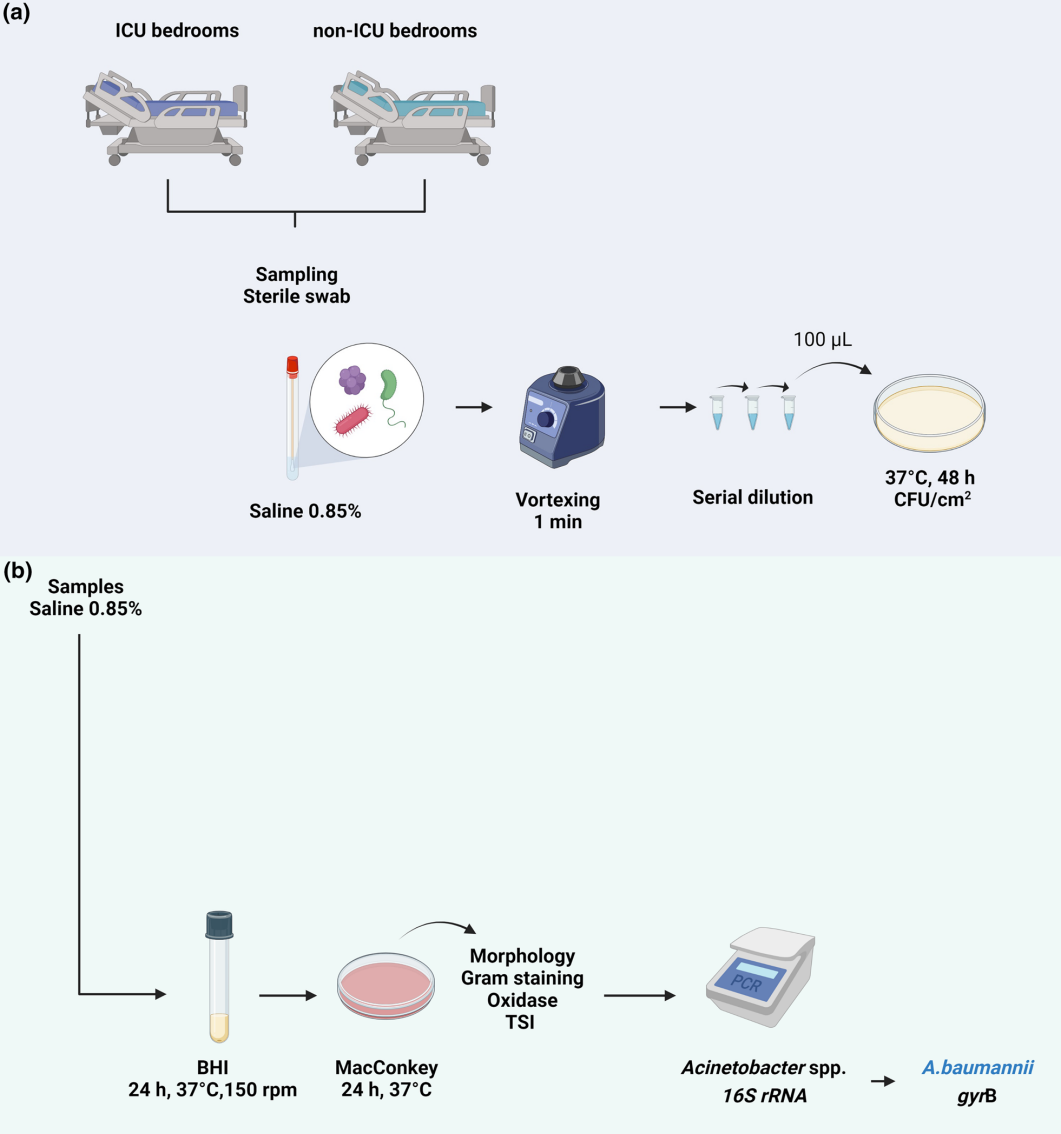
Flowchart of uncoated and copper-coated surface sampling on hospital bedrooms for total aerobic bacterial loads counting (**A**) and isolation and identification of *Acinetobacter* spp. (**B**)

Samples from the hospital environment were collected weekly with sterile swabs moistened with 0.85% saline and rolled over each surface containing copper-coated films and the equivalent area on the surfaces without films. Samples from the four bed rails, two bed cranks, two and three light switches in ICU and non-ICU, respectively, and three over-bed tray tables of the same bedroom were grouped forming a pool by kind of sample. All the samples in saline were vortexed for 1 min, serially diluted to 10^–2^ and 100-µL aliquots were spread on Plate Count Agar (PCA; Merck KGaA, Germany), with 48 h of incubation at 37 °C for colony counting. All bacterial counts were expressed as CFU/cm^2^ corresponding to sampled area (Fig. 2A).

### Isolation and identification of Acinetobacter spp.

Swabs used to collect samples from the described surfaces were transferred from 0.85% saline tubes to 3 mL of BHI broth and incubated for 24 h at 37 °C under shaking at 150 rpm. Subsequently, samples were spread on MacConkey agar (Oxoid, England) and incubated for 24 h at 37 °C. All assays were performed in duplicate. *Acinetobacter* spp. colonies were screened by presumptive characteristics of morphology and evaluated by Gram staining, oxidase (Laborclin, Brazil) and Triple Sugar Iron (TSI; Oxoid, England) tests. Isolates presenting a profile compatible with *Acinetobacter* spp. were submitted to DNA extraction by heating (Donald et al. 2000), followed by PCR targeting the *16S rRNA* gene to confirm the genus *Acinetobacter* (Turton et al. 2005) using the following conditions: initial denaturation at 95 °C for 3 min, followed by 30 cycles of denaturation at 95 °C for 30 s, annealing at 48 °C for 35 s and extension at 72 °C for 35 s, with a final extension at 72 °C for 7 min. The identification of *A. baumannii* was confirmed by multiplex PCR targeting *gyr*B (Higgins et al. 2007). The conditions used for the amplification of *gyr*B were the same employed for the *16S rRNA* gene, but with annealing temperature at 54 ºC. Universal primers targeting bacterial *16S rRNA* gene were employed in the reactions as internal control to demonstrate that no inhibition has occurred (Higgins et al. 2004) (Fig. 2B). DNA from *A. baumannii* ATCC 17978 and a DNA-free reaction were used as positive and negative controls, respectively.

### Antimicrobial susceptibility testing

Antimicrobial susceptibility of *A. baumannii* and *A. nosocomialis* strains isolated from copper-coated films and control surfaces was evaluated by the disk diffusion method according to the Clinical and Laboratory Standards Institute (CLSI) guidelines, and results were interpreted following CLSI criteria (CSLI 2018). The antimicrobials tested were: amikacin (AMI), ampicillin-sulbactam (APS), cefepime (CPM), cefotaxime (CTX), ceftazidime (CAZ), ceftriaxone (CRO), ciprofloxacin (CIP), doxycycline (DOX), gentamicin (GEN), imipenem (IPM), levofloxacin (LEV), meropenem (MPM), piperacillin-tazobactam (PIT), sulfamethoxazole-trimethoprim (SUT), tetracycline (TET), ticarcillin-clavulanic acid (TAC), and tobramycin (TOB) (DME, Brazil).

The strains were also evaluated regarding the minimum inhibitory concentration (MIC) of polymyxin B (EDQM, France) determined by the broth microdilution method and results were interpreted according to the CLSI criteria (CSLI 2018). *Escherichia coli* ATCC 25922 and *Pseudomonas aeruginosa* ATCC 27853 were used as reference strains for antibiotic quality control. Strains that were not susceptible to at least one agent in ≥ three antimicrobial categories were defined as MDR (multidrug resistant); absence of susceptibility to at least one agent in all but ≤ two categories was defined as XDR; and PDR (pan drug resistance) was defined as the absence of susceptibility to all tested antimicrobials (Magiorakos et al. 2012).

### Detection of *bla*_OXA-23_ and *bla*_NDM_ genes in *A. baumannii*

Carbapenem-resistant *A. baumannii* (CRAb) were assessed by PCR for the presence of the *bla*_OXA-23_ and *bla*_NDM_ genes as previously described (Donald et al. 2000; Fallah et al. 2014). Amplification conditions used for the *bla*_OXA-23_ gene were: initial denaturation at 95 °C for 5 min, followed by 30 cycles of 95 °C for 30 s, 58 °C for 45 s, and 72 °C for 1 min 30 s, and a final extension step at 72 °C for 7 min. Amplification of the *bla*_NDM_ gene was carried out with the following conditions: initial denaturation at 95 °C for 3 min, followed by 35 cycles of at 95 °C for 20 s, and 57 °C for 30 s, 72 °C for 40 s, and a final extension at 72 °C for 7 min. DNA from a clinical isolate previously identified as carrier of *bla*_OXA-23_ and *bla*_NDM_ was used as positive control and a DNA-free reaction as negative control.

### Statistical analysis

After total aerobic bacterial counting on PCA agar plates, the mean bacterial counts of each surface containing copper-coated films and control surfaces were determined and compared to evaluate the influence of copper films on bacterial counts. The data were assessed by parametric t-Test using GraphPad Prism software version 8.2.0 (GraphPad Software, USA), and the level of significance was set to 0.05.

## Results

### In vitro antimicrobial efficacy of adhesive copper films

**Fig. 3:**
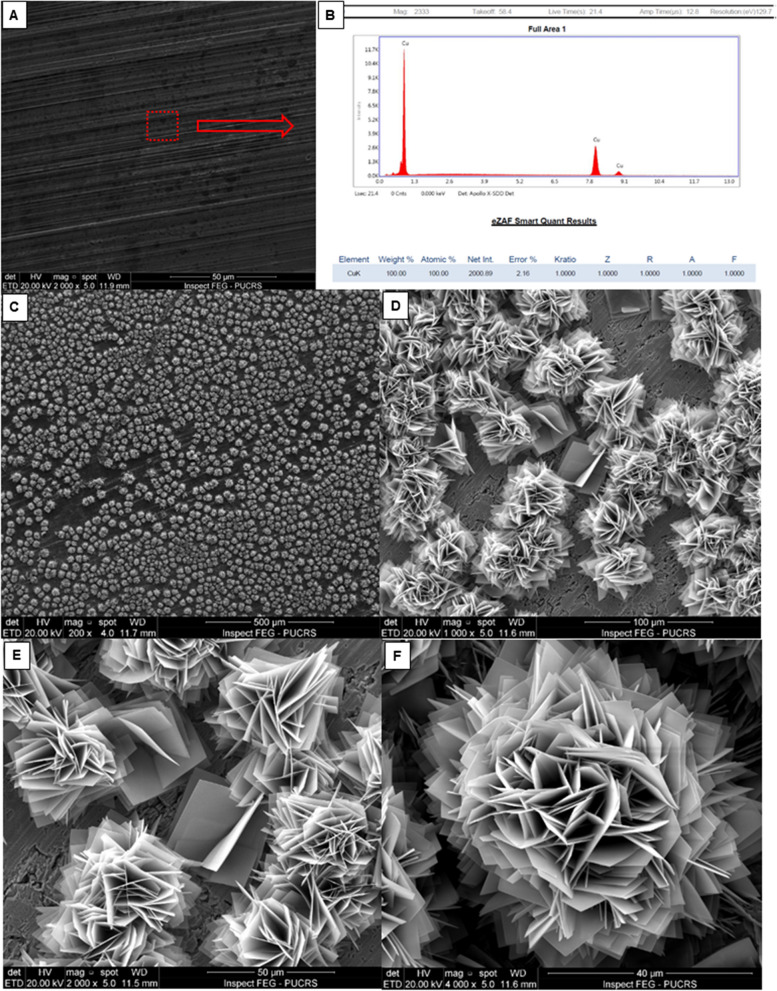
Copper film surfaces observed by scanning electron microscopy. Copper film without artificial contamination (**A**) and respective energy-dispersive X-ray spectroscopy (EDS) spectra and elemental mapping of copper (**B**). Copper film after exposure to *Acinetobacter baumannii*, with no presence of bacterial cells and flower-like structure in oxidized copper film at different magnifications (**C**, 200X; **D**, 1000X; **E**, 2000X; and **F**, 4000X)

Adhesion and biofilm formation of the *A. baumannii* reference strain and clinical isolate were inhibited to below the detection limit (10^1^ CFU/mL) on copper-coated films, since neither bacterial presence was detected on SEM analysis (Fig. 3), nor bacterial growth was observed on plate counting.

The SEM images showed nanosheets of oxidized copper disposed along specific directions and able to interlace and overlap with each other forming a flower-like structure. The energy-dispersive X-ray spectroscopy (EDS) elemental analysis confirmed the presence of only copper in the films (Fig. 3).

### Evaluation of antimicrobial action of copper adhesive films in hospital environment

**Fig. 4:**
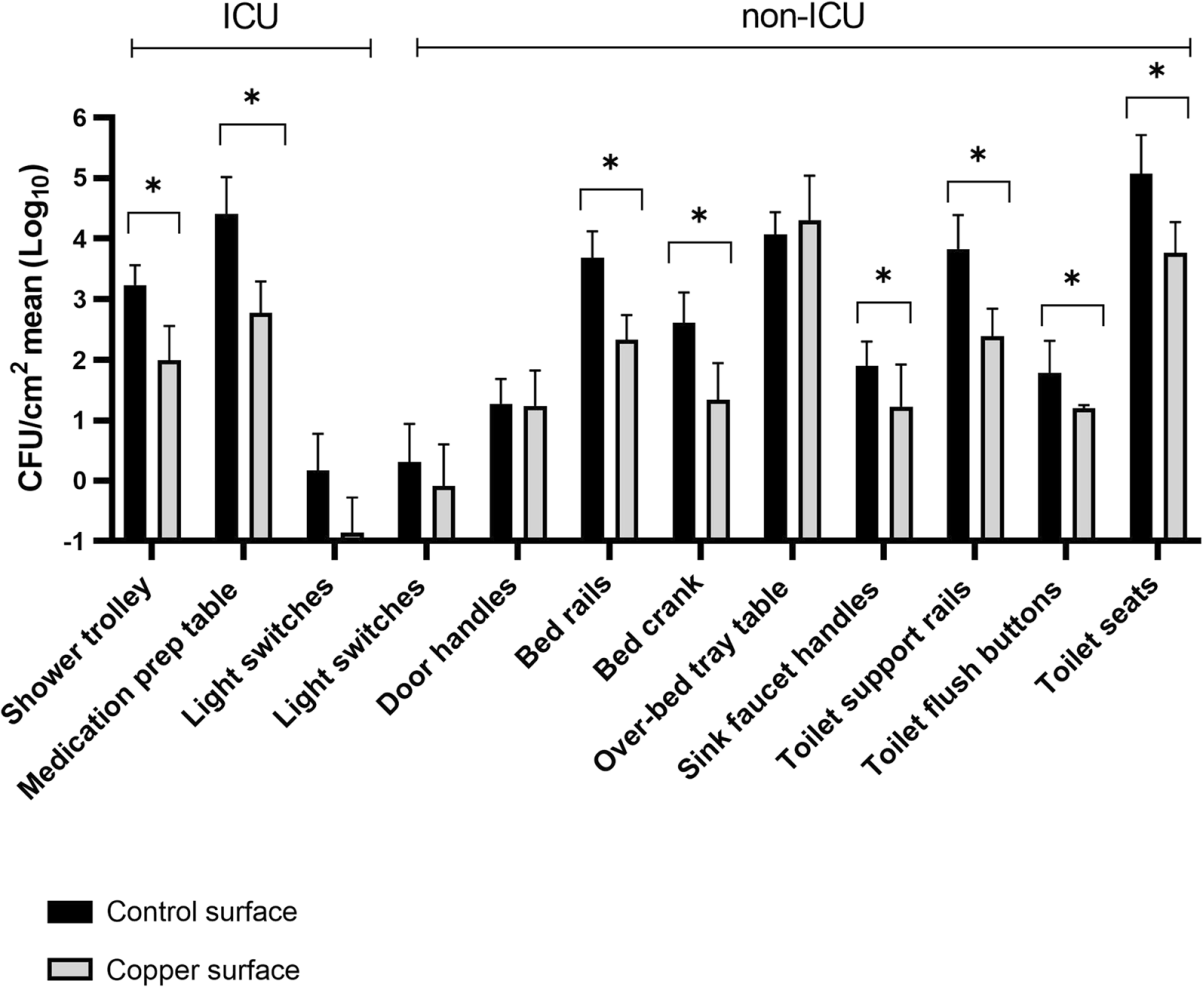
Bacterial loads from highly touched surfaces coated with copper film and their uncoated counterparts collected from December 2018 to May 2019, expressed by the mean of CFU/cm^2^. ICU: Intensive Care Unit. *indicates significant difference (P-values ≤ 0.05) as copper and control surfaces were compared

A total of 1,632 samples were collected during the six months of the study. Highly touched hospital surfaces coated with copper film showed lower bacterial counts when compared to control surfaces at the following places: shower trolley (ICU), medication prep table (ICU), bed rails, bed crank, toilet support rails, toilet flush buttons, toilet seats, and sink faucet handles (Fig. 4). In contrast, the over-bed tray table, door handles, ICU and non-ICU light switches did not show significant differences in bacterial counts among the samples (Fig. 4).

### Acinetobacter spp. in the hospital environment

**Fig. 5:**
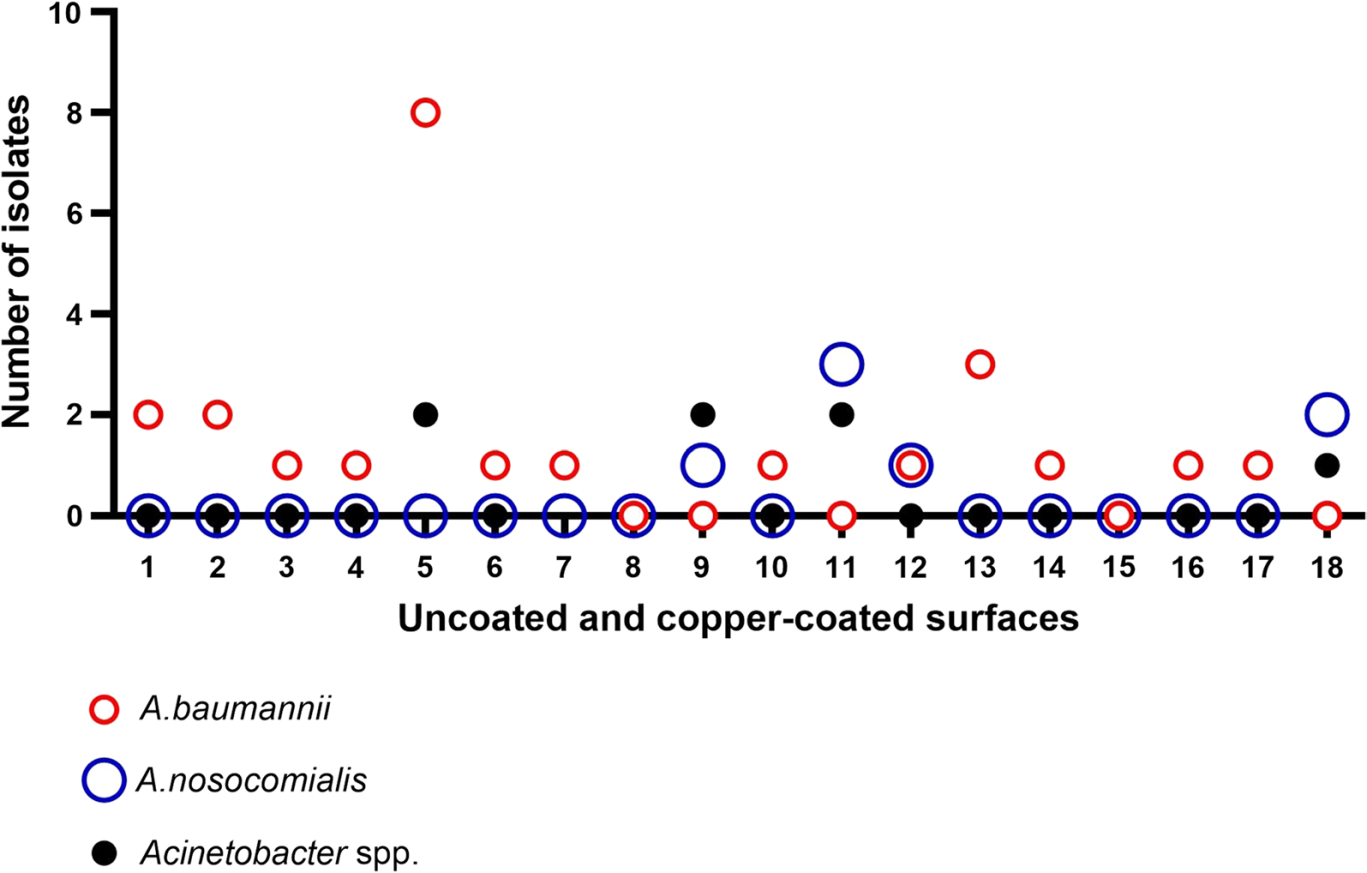
*Acinetobacter* spp. isolated from uncoated and copper-coated surfaces in the hospital environment. Even numbers indicate uncoated surfaces and odd numbers indicate copper-coated surfaces. Medication prep table uncoated and copper-coated (1 and 2, respectively), light switches (3 and 4), bed rails (5 and 6), bed crank (7 and 8), light switches (9 and 10), over-bed tray table (11 and 12), toilet support rails (13 and 14), sink faucet handles (15 and 16), and toilet seats (17 and 18)

*Acinetobacter* spp. were isolated in 39 samples (2.4%) from hospital surfaces during the six months of collection. Of these, 28.2% were recovered from the copper-coated surfaces and 71.8% from control surfaces (Fig. 5). *Acinetobacter* spp. were not detected in shower trolley, door handles and toilet flush buttons either covered or not with copper film. All isolates were presumptively identified by biochemical tests and had their identity confirmed as *Acinetobacter* spp. by PCR targeting the *16S rRNA* gene. Among the 39 *Acinetobacter* spp. isolates, 24 (61.54%) were identified at species level as *A. baumannii* and seven (17.95%) as *A. nosocomialis* by *gyr*B multiplex PCR. Bed rails were the site that presented the highest number of *A. baumannii* isolates. *A*. *nosocomialis* was found in only three sites: light switches from non-ICU, over-bed tray table and toilet seats.

*A. nosocomialis* isolated from hospital surfaces presented susceptibility to most antimicrobials, but six isolates presented intermediate or full resistance to ceftriaxone, four to cefotaxime, two to tetracycline and one to trimethoprim-sulfamethoxazole (from control surfaces only). By contrast, 13 (54.17%) *A. baumannii* isolates showed reduced susceptibility to most antimicrobials. All these 13 isolates proved to be resistant to carbapenems and classified as XDR, being four of them isolated from copper surfaces (light switches from ICU, one; medication prep table from ICU, two; over-bed tray table from non-ICU, one) and nine from control surfaces (light switches from ICU, one; medication prep table from ICU, two; toilet support rails, one; bed rails, five).

All *A. baumannii* and *A*. *nosocomialis* isolates were susceptible to polymyxin B. Among the carbapenem-susceptible isolates, the MIC values for polymyxin B ranged from 0.25 µg/mL to 1.0 µg/mL, whereas CRAb isolates presented MIC values ranging from 0.5 µg/mL to 2.0 µg/mL. These isolates were evaluated for the presence of the *bla*_OXA-23_ and *bla*_NDM_ genes. The *bla*_OXA-23_ gene was present in all CRAb isolates, but none of them harbored the *bla*_NDM_ gene.

## Discussion

HAIs are an escalating concern that constantly challenges IPC measures on healthcare facilities (Michels et al. 2015), especially when it involves multi-drug resistant bacteria that are able to survive for long periods on abiotic surfaces (Kramer et al. 2006). Highly-touched hospital surfaces can be a hot spot for bacterial transmission to patients (Boyce 2007) and a wide variety of strategies to mitigate this problem have been investigated. Surface coating with antibacterial properties is one of these strategies. The coating of surfaces and equipment with copper films can play an important role in reducing contamination due to its antimicrobial effectiveness to kill different microorganisms and, consequently, decrease the risk of HAIs (Kruk et al. 2015; Michels et al. 2015).

Copper-coated films have shown antibacterial action both in laboratory assays and in the nosocomial environment monitored over six months, although the reduction pattern observed has been shown to be stronger on in vitro analysis. This can be partially explained by the copper oxidation nature. The 99.9% metallic copper-coated films in aqueous solution, such as the medium culture used, is easily oxidated to cuprous (Cu^+^) and cupric (Cu^2+^) states (Lemire et al. 2013; Popov et al. 2020). These states can be confirmed by SEM images, which enables the visualization of nanosheets of oxidized copper. Since mono and divalent forms of copper are toxic to bacteria (Mikolay et al. 2010), no cellular growth was observed for the two evaluated *A. baumannii* strains (*A. baumannii* ATCC 17978 and the XDR *A. baumannii* Acb-220). Biofilm formation by *A. baumannii* was also inhibited when copper was present, probably in early stages of the bacterial adherence process, as already observed by a study evaluating *S*. *aureus* adhesion in polyelectrolite multilayers with copper nanoparticle films (Kruk et al. 2019).

On the contrary to what was observed in vitro, the analyzes performed on nosocomial environment demonstrated the presence of bacteria on copper-coated surfaces, although in reduced loads than those found in the uncoated controls. Since the copper oxidation process is relatively slower when in contact with air than in aqueous solution, coated films present a higher content of metallic copper that shows slightly lower toxicity when compared to the mono and divalent ionic states. Furthermore, surfaces on hospital bedrooms are frequently touched and contaminated (Mikolay et al. 2010). Nevertheless, from a total of 1,632 samples collected during the six months of the study, only 39 *Acinetobacter* spp. isolates were recovered predominantly from control surfaces without copper-coated films. Twenty-four isolates were identified as *A. baumannii*, seven as *A. nosocomialis* and eight as other species from the genus *Acinetobacter*.

A significant reduction of bacterial counts was observed on medication prep table and shower trolley surfaces containing the copper-coated films on ICU bedrooms. Light switches also presented a decrease in bacterial load, yet discreet, in comparison with uncoated control surfaces. From all surfaces usually linked to HAIs, bed rails are reported among the most contaminated sites in hospital bedrooms, since these rails are constantly in close contact to patients and are recurrently touched by hospital staff and visitors (Raro et al. 2017; Boyle et al. 2019). In this study, a 95.5% reduction of total aerobic bacterial load on copper-coated films was observed on bed rails, which was similar to the findings reported by Schmidt and colleagues (Schmidt et al. 2020). A higher number of *A. baumannii* contaminated samples was recovered from uncoated surfaces when compared to copper-coated surfaces (eight and one, respectively) on bed rails of non-ICU bedrooms. In fact, *A*. *baumannii* sampled from bed rails has already been associated with an outbreak in a medical-surgical ICU by Catalano and colleagues (Catalano et al. 1999). These data reinforce the danger posed by the persistence of this pathogen in the hospital environment, as well as highlight bed rails as one of the possible reservoirs of strain transmission to patients. The use of copper films as an effective strategy to prevent bed rails as reservoirs of this pathogenic agent must be considered in the IPC protocols design.

All *A*. *baumannii* isolates, regardless of the surface sampled, were resistant to carbapenems, which was already expected because of the high rates of resistance to these antimicrobials being reported worldwide (Gales et al. 2019; Ayobami et al. 2020; Jernigan et al. 2020). Production of carbapenemases is the most frequently observed and worrisome mechanism of resistance to carbapenems present in *A*. *baumannii* (Ramirez et al. 2020). Herein, we detected the *bla*_OXA-23_ gene in all isolates, which had already been described in *A*. *baumannii* isolates recovered from the same hospital (Raro et al. 2017). Indeed, OXA-23 is the most widely disseminated class D-carbapenemase, being present in all countries of Latin America (Rodríguez et al. 2018). On the other hand, *bla*_NDM_ was not detected, as also reported in a study conducted on another Brazilian hospital (Romanin et al. 2019). Despite the reports of an increased incidence of polymyxin-resistant *A*. *baumannii*, partially due to the resurgence of polymyxin prescription against carbapenem-resistant gram-negative bacteria (Lima et al. 2020), all isolates recovered in this study fortunately presented a susceptible phenotype.

Although *A*. *baumannii* is the most prevalent *Acinetobacter* species found in hospital surfaces, non-*A. baumannii* species are increasingly being recognized as nosocomial pathogens presenting the same ability to cause outbreaks and acquire resistance to multiple antimicrobials (Park et al. 2013). Therefore, we also investigated the presence of non-*A. baumannii* species and antimicrobial susceptibility of *A. nosocomialis* isolated from hospital surfaces. Among all isolates, 38.5% were identified as non-*A. baumannii* species, with *A. baumannii* isolates presenting higher levels of resistance.

It is known that bacteria can physiologically adapt to metal stress by different mechanisms and withstand metal toxicity (Lemire et al. 2013). On HAIs scenario, this could be an important drawback, since co-selection for antibiotic resistance can be observed among bacteria exposed to metals such as copper (Wales and Davies 2015). However, coating highly touched surfaces with copper apparently did not co-select antibiotic resistance. In fact, all *A*. *baumannii* isolates detected were classified as XDR, and most of them were isolated from uncoated control surfaces. Furthermore, *A*. *nosocomialis* isolates were susceptible to the majority of the analyzed antimicrobials regardless of being recovered from copper-coated or uncoated surfaces.

These findings suggest that highly touched surfaces covered with copper films in ICU and non-ICU bedrooms were capable of contributing to IPC measures reducing total aerobic bacterial loads, including *Acinetobacter* spp. In addition, *A*. *baumannii* XDR isolates were more prevalent on uncoated surfaces versus copper-coated surfaces, suggesting that copper does not exert co-selection for resistance to antibiotics at least in the context of this study.

## Data Availability

The datasets used and/or analyzed in this study are available from the corresponding author upon reasonable request. All the digital illustrations were created with BioRender under Academic License Terms.

## Abbreviations

HAIs: Healthcare-associated infections
ICU: Intensive care unit
IPC: Infection prevention and control
ATCC: American type culture collection
BHI: Brain heart infusion
CFU: Colony-forming units
PBS: Phosphate-buffered saline
SEM: Scanning electron microscopy
LabCeMM: Central laboratory of microscopy and microanalysis
PCA: Plate count agar
TSI: Triple sugar iron
PCR: Polymerase chain reaction
CLSI: Clinical and laboratory standards institute
MIC: Minimum inhibitory concentration
MDR: Multidrug resistant
XDR: Extreme drug resistance
PDR: Pan drug resistance
EDS: Energy-dispersive X-ray *spectroscopy*
SD: Standard deviation
CRAb: Carbapenem-resistant *A. baumannii*
